# Suicide prevention: Precision suicidology is needed

**DOI:** 10.1192/j.eurpsy.2024.31

**Published:** 2024-08-27

**Authors:** P. Courtet

**Affiliations:** University of Montpellier, Montpellier, France

## Abstract

**Abstract:**

Suicidal behavior is a public health challenge that resists the various efforts made toward its prevention and treatment. Indeed, suicide rate have not significantly changed in the past decades. Then one may wonder if precision psychiatry could be the solution?

Advances towards precision suicidology will be detailed from detection to oportunities for treatment.

First, current suicide risk assessment methods are unable to detect suicidal risk with sufficient accuracy and while thousand of risk factors for suicide have been identified, they are no more accurate in predicting suicidal behavior than flipping a coin. Second, we are lacking specific and effective evidence based strategies for suicide prevention.

The aim of precision psychiatry is tailoring efficient preventive and therapeutic approaches to the unique characteristics of each patient. It assumes that the determination of a reliable medical diagnosis is unfeasible if based on symptomology alone and it must integrate genomics data, clinical dimensions, biomarkers, and environmental and lifestyle factors and this amount of data analysed by artificial intelligence would give us “biosignatures” that would yield a more appropriate diagnosis, treatment and prognosis.

We will cover advances in genomics, imaging, inflammatory markers and digital health that witness the realistic possibility to change the field of suicide prevention.

**Disclosure of Interest:**

None Declared

